# Terahertz Reconfigurable Intelligent Surfaces (RISs) for 6G Communication Links

**DOI:** 10.3390/mi13020285

**Published:** 2022-02-10

**Authors:** Fengyuan Yang, Prakash Pitchappa, Nan Wang

**Affiliations:** Institute of Microelectronics, Agency for Science, Technology and Research, Singapore 138634, Singapore; yang_fengyuan@ime.a-star.edu.sg

**Keywords:** reconfigurable intelligent surface (RIS), terahertz (THz), 6G communication, reconfigurable metasurface

## Abstract

The forthcoming sixth generation (6G) communication network is envisioned to provide ultra-fast data transmission and ubiquitous wireless connectivity. The terahertz (THz) spectrum, with higher frequency and wider bandwidth, offers great potential for 6G wireless technologies. However, the THz links suffers from high loss and line-of-sight connectivity. To overcome these challenges, a cost-effective method to dynamically optimize the transmission path using reconfigurable intelligent surfaces (RISs) is widely proposed. RIS is constructed by embedding active elements into passive metasurfaces, which is an artificially designed periodic structure. However, the active elements (e.g., PIN diodes) used for 5G RIS are impractical for 6G RIS due to the cutoff frequency limitation and higher loss at THz frequencies. As such, various tuning elements have been explored to fill this THz gap between radio waves and infrared light. The focus of this review is on THz RISs with the potential to assist 6G communication functionalities including pixel-level amplitude modulation and dynamic beam manipulation. By reviewing a wide range of tuning mechanisms, including electronic approaches (complementary metal-oxide-semiconductor (CMOS) transistors, Schottky diodes, high electron mobility transistors (HEMTs), and graphene), optical approaches (photoactive semiconductor materials), phase-change materials (vanadium dioxide, chalcogenides, and liquid crystals), as well as microelectromechanical systems (MEMS), this review summarizes recent developments in THz RISs in support of 6G communication links and discusses future research directions in this field.

## 1. Introduction

**Figure 1:**
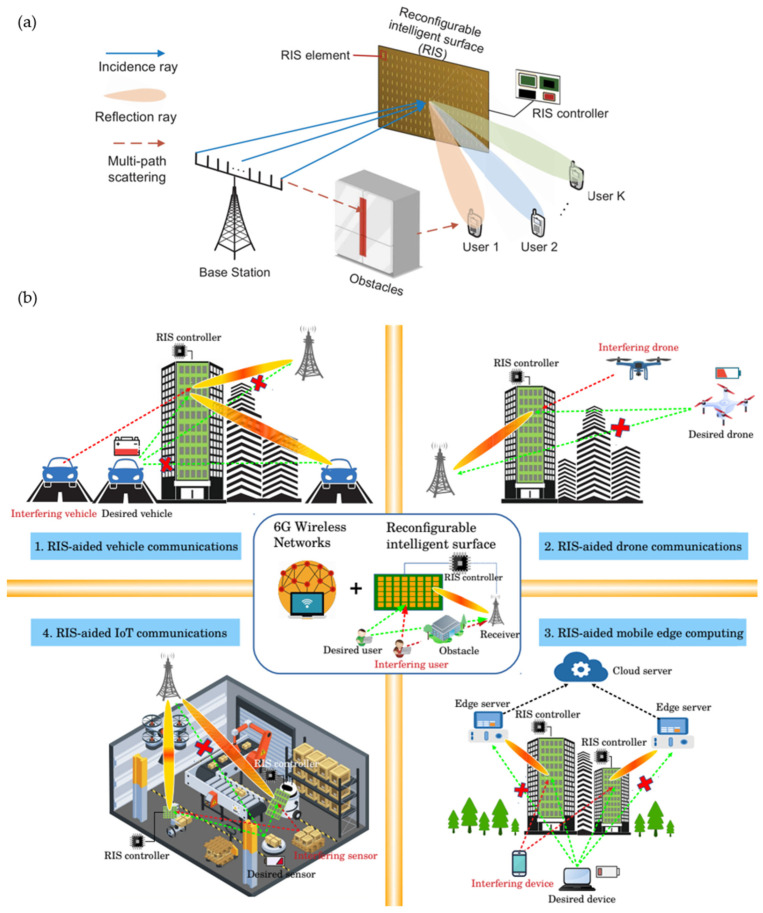
RIS-assisted wireless communication. (**a**) RIS-enabled non-line-of-sight (NLOS) transmission. Reprinted from Ref. [17]. (**b**) RIS-embedded smart infrastructures for future 6G communications. Reprinted from Ref. [18].

With the commercial launch of the fifth generation (5G) network in 2020, research into the sixth generation (6G) communication system is eagerly on the agenda [1,2,3]. In order to meet the communication requirements of modern society in building smart cities, the 6G wireless network is envisioned to provide ultra-fast data rates (~1 Tbps), ultra-low latency (<1 ms), ubiquitous wireless connectivity, superior spectral and energy efficiency, as well as extremely high reliability and security [4,5,6]. The terahertz (THz) spectrum, ranging from 0.1 THz to 10 THz, is well suited for 6G applications due to its higher frequency, large bandwidth, and short response time [7,8,9]. However, the THz spectrum is highly absorbed by water molecules in the atmosphere, and its ultra-short wavelength is easily scattered by obstacles along the propagation path. This high path loss limits the THz wave to short-range transmission. Energy-concentrated directive beam transmission is imperative for the THz network to compensate for propagation losses. In contrast with active phased-array antennas [10,11] applied on the transmitting and receiving end, reconfigurable intelligent surfaces (RISs) are proposed to modify the wireless transmission path [12,13,14,15,16]. RIS consists of periodic elements (meta-atoms or unit cells) altering the phase and magnitude of incident waves to have constructive/destructive interference in desired directions. Based on this, THz wireless communication is capable of being extended to non-line-of-sight (NLOS) transmission (Figure 1a) [17]. Compared with existing relays using power amplifiers for wave receiving and retransmitting, RIS enhances the transmission by phase control using nearly passive elements, requiring less spectrum and energy [4,13]. Therefore, RISs can be embedded within infrastructure cost-effectively, enabling advanced wireless communication in 6G smart cities (Figure 1b) [18]. RIS is also known as reprogrammable intelligent surface [19,20] or intelligent reflecting surface (IRS) [21,22] in some contexts. The latter primarily denotes an intelligent surface operating in the reflective mode. A promising application of such a surface is deploying it on infrastructure to reflect waves in a desired direction [4].

RIS benefits have been realized as a result of the flourishing development of electromagnetic metasurfaces in the last decade. Metasurfaces are two-dimensional versions of metamaterials and have the advantages of low profile, reduced loss, and ease of fabrication [3,4]. Metasurfaces consist of artificially designed periodic structures, enabling unprecedented wave manipulation that is unattainable with natural materials [23]. In 2011, Yu et al. first proposed the generalized Snell’s law, including abrupt phase shifts from the metasurface unit cells to realize nearly arbitrary wavefront shaping [24]. Since then, various fascinating wavefront manipulations have been demonstrated, such as reflectionless wide-angle refraction [25,26], sub-diffraction focusing with ultrathin planar lens [27,28], and wave-impedance matching across different material boundaries [29]. Such metasurfaces have only a static function once fabricated, whereas reconfigurable metasurfaces with tunable functions are gaining attention. The reconfigurable function is achieved by incorporating meta-atoms with tunable components or materials, which alter the electromagnetic response through external stimuli. With the development of digital control, coding metasurfaces were proposed to manipulate waves by changing the coding sequences of digital particles (unit cells) [30,31,32,33,34,35,36]. Active electronic components, such as positive-intrinsic-negative (PIN) diodes [30,37,38,39], have been widely applied in RISs working at 5G networks but are incompetent for applications in 6G systems due to the cutoff frequency limit. To overcome this challenge, reconfigurable technology approaches from both the electronic and optical sides are being explored intensively to fill the “THz gap” [40]. Beyond that, phase-change materials and microelectromechanical systems (MEMS) for THz RISs are also widely investigated.

Most existing review papers focused on summarizing various possible applications of RISs in wireless communication [41] or analyzing different tunable metasurfaces according to their tuning mechanisms [42,43]. Several review papers analyzed active metasurfaces for only one tuning element [44,45,46,47]. This paper emphasizes reconfigurable metasurfaces operating in the THz spectrum with the potential to assist 6G communications, i.e., metasurfaces with tunable functions such as pixel-level amplitude modulation or wide-range phase coverage for beam steering. Polarization modulation is briefly discussed as a possible future carrier-wave function. To underline the reconfigurability, RISs are categorized based on their tuning mechanisms, including complementary metal-oxide-semiconductor (CMOS) transistors, Schottky diodes, high-electron mobility transistors (HEMTs), graphene, photoactive semiconductor materials, phase-change materials (vanadium dioxide, chalcogenides, and liquid crystals), and MEMS.

The rest of this paper is organized as follows: Section 2 presents various reconfigurable metasurfaces with pixel-level amplitude modulation or dynamic-beam-steering functions at THz frequency (0.1 THz–10 THz). Subsections are divided according to the tuning elements. Section 3 summarizes the properties of THz RISs and discusses possible future research directions.

## 2. Terahertz Reconfigurable Intelligent Surface 

Various THz RISs with the potential to assist 6G communications are discussed in this section. The functionality of the reconfigurable metasurface focuses on amplitude modulation at the pixel-level or phase modulation for dynamic beam steering. The subsections are devoted to the tuning elements, investigating the reconfigurable mechanism for achieving RIS. The analysis begins with electronic approaches (CMOS transistors, Schottky diodes, HEMTs, and graphene), followed by optical approaches involving semiconductor materials, then phase change materials (vanadium dioxide, chalcogenides, and liquid crystals), and finally MEMS-based structural deformation.

### 2.1. Electronic Approaches

Active electronic components, such as diodes and varactors, have been widely utilized in microwave and millimeter-wave regimes for reconfigurable metasurface control [30,37,38,39,48,49,50,51]. However, they are incompatible with 6G applications in the THz spectrum due to the restricted cutoff frequency and dramatically increased loss [52]. To overcome this obstacle and implement reconfigurable metasurfaces in the THz spectrum using electronic approaches, CMOS transistors have to be embedded in meta-atoms with specially designed structures for local resonances [52]. Alternative solutions include integrating metasurface meta-atoms with layered semiconductor structures or graphene with electrical tunability.

#### 2.1.1. Complementary Metal-Oxide-Semiconductor (CMOS) Transistor

**Figure 2:**
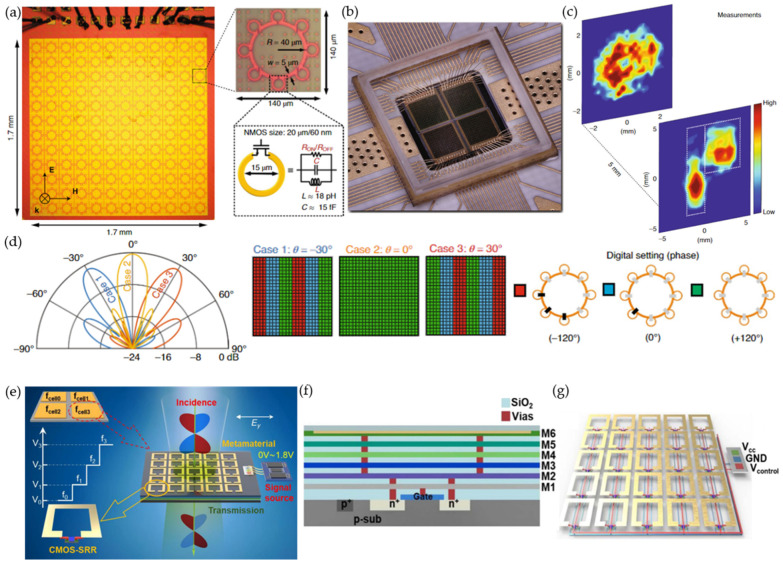
CMOS transistor-enabled reconfigurable metasurface. (**a**–**d**) GHz-speed programmable metasurfaces using CMOS-based chip tiles. (**a**) A single silicon chip tile consists of a 12 × 12 array (left). The enlarged portion (right) shows the unit-cell structure with active NMOS transistors embedded in the gap of inductive microloops. Each unit cell has an eight-bit control, enabling 256 states for amplitude and phase control. (**b**) Photo of the fabricated 2 × 2 tiled chips, which were wire-bonded to a customized printed circuit board for external voltage control. (**c**) Amplitude modulation was experimentally demonstrated as a holographic projection of the letter ‘P’. (**d**) Beam steering at ±30° with the corresponding three different phase profiles and meta-element digital settings. Reprinted from Ref. [52]. (**e**–**g**) Reconfigurable metasurface based on CMOS structures. (**e**) A bias voltage is applied for transmitted amplitudes and phase modulation. The reconfigurable metamaterial can be divided into subsections for greater functionality. (**f**) Cross section of the unit cell, consisting of six layers for the CMOS transistor configuration. (**g**) Layout with wire connection for the biasing control. Reprinted from Ref. [53].

Incorporating active electronic devices into a passive metasurface allows for fast dynamic control, but the application is limited to microwave frequencies. In 2020, Venkatesh et al. presented a programmable metasurface using complementary metal-oxide-semiconductor (CMOS) transistors operating at 0.3 THz beyond its cutoff frequencies [52]. They applied a 65 nm industry-standard CMOS process to fabricate the metasurface in a silicon chip tile, consisting of 12 × 12 arrays (Figure 2a). Each meta-atom contained eight n-type metal-oxide-semiconductor (NMOS) transistors for an eight-bit reconfiguration at gigahertz (GHz) speed. Parallel subwavelength inductive microloops were added to the transistor for local resonance to suppress the non-negligible parasitic capacitance leakage. An amplitude modulation of 25 dB was achieved between all switches open (maximum transmission) and closed (minimum transmission) states. A demonstration of the 2 × 2 tiled chips (Figure 2b) for holographic projections of the letter ‘P’ is shown in Figure 2c. The unit-cell structure was derived from a general C-shaped split-ring resonator (SRR), which controls the transmitted amplitude and phase by varying the gap-opening orientation and size, respectively. This was realized by selectively switching the eight transistors of a meta-atom, leading to 256 states in total. Due to the symmetric configuration, each meta-atom had 84 unique codes and realized a phase coverage of 260°. Beam steering from 0° to ±30° was achieved by configuring the unit cell in three different phase profiles and meta-atom digital settings (Figure 2d).

By utilizing custom-designed CMOS-based semiconductor structures, it is possible to circumvent the cutoff frequency limitation of commercial transistors (Figure 2e) [53]. The meta-atom structure, consisting of a square SRR on top of a six-layer CMOS-based semiconductor structure, is shown in Figure 2f. The gap of the SRR was connected to the source and drain of a metal-oxide-semiconductor field-effect transistor (MOSFET) through vias (Figure 2g). The metasurface was fabricated with 180 nm CMOS technology. With a bias voltage from 0 V to 1.8 V, a redshifted frequency of 35 GHz and a phase difference of 3° were achieved at 0.3 THz. Although the phase modulation was limited, the authors presented an alternative solution for realizing a CMOS-based reconfigurable metasurface.

#### 2.1.2. Schottky Diode

**Figure 3:**
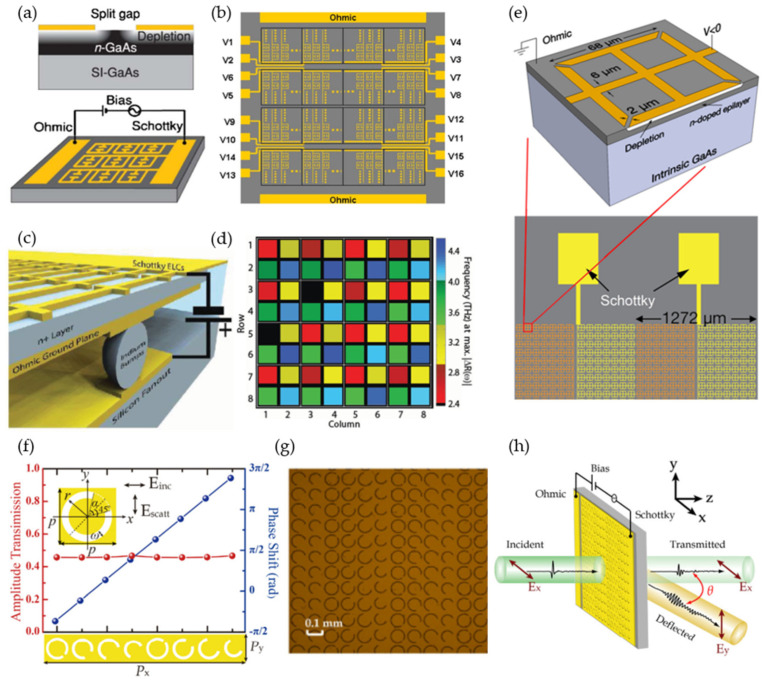
Schottky-diode-structure-enabled reconfiguration. (**a**,**b**) A 4 × 4 pixel amplitude modulator. (**a**) Schematic of the cross section of the unit cell incorporating SRR with Schottky gate structure (top). The gray scale of the depletion region indicates the free charge-carrier density. A single pixel on the THz SLM for amplitude modulation (bottom). (**b**) THz SLM consisting of 4×4 pixels. Reprinted from Ref. [55]. (**c**,**d**) An 8 × 8 four-color spatial light modulator. (**c**) Schematic of the metamaterial absorber with a flip-chip-bonded, n-doped GaAs epitaxial layer. (**d**) An example of the spatial light modulator with different frequencies for each pixel. Reprinted from Ref. [56]. (**e**) A diffractive modulator with grating configuration realizing 22 dB amplitude modulation at 36.1°. Reprinted from Ref. [57]. (**f**–**h**) A phase-modulated deflector. (**f**) An array consisting of eight unit cells realized 2π phase control with nearly the same transmission efficiency. (**g**) Microscopic image of the fabricated metasurface. (**h**) An illustration of the deflected wave transmission. Reprinted from Ref. [58].

Commercial off-the-shelf (COTS) diodes have been extensively used in 5G RIS for active tuning; the concept was adopted in the THz spectrum by constructing semiconductor metamaterials with a Schottky gate structure. This idea was first realized by an active metamaterial switch in 2006 [54]. The Schottky junction was formed by integrating a metallic SRR with a 1 μm thick n-doped gallium arsenide (GaAs) layer (Figure 3a) [55]. Applying a reverse gate-bias voltage alters the substrate charge-carrier density around the split gap, thus affecting the resonance response of the SRRs. Later, the same group used the unit cell to build a 4×4 pixelated spatial light modulator (Figure 3b). Each pixel, consisting of 50×50 split-ring resonators (SRRs) with a total size of 4×4 ${\mathrm{mm}}^{2}$, was independently controlled by the external bias voltage. An amplitude-modulation depth of ~3 dB was achieved at 16 V bias voltage in kilohertz (kHz) speed. The modulation speed was enhanced to megahertz (MHz) by placing the ohmic ground plane directly underneath the Schottky layer to minimize the device capacitance, as shown in Figure 3c [56]. This was utilized to build a four-color, 8×8 pixelated spatial light modulator by repeating a 2 × 2 four-color subarray, thereby realizing a more advanced spatial and spectral control (Figure 3d).

To realize beam steering, a switchable-diffraction grating with combined amplitude and phase modulation using Schottky gate structure for tuning was presented in [57] (Figure 3e). By applying a reverse bias voltage (−13 V) on alternate columns, the metasurface achieved 20 dB amplitude modulation at 36.1° with a speed of 1 kHz at 0.4 THz. An alternative method applied to achieve beam steering was shown using meta-atoms with specially designed ‘C’-shaped structures covering a 2*π* phase range. Figure 3f shows eight ‘C’-shaped SRRs with different gap sizes and orientations for a π/4 phase gradient [58]. The metasurface metallic pattern was made using gold film and embedded with a doped semiconductor substrate (Figure 3g) [58]. As a result, the metasurface realized broadband (0.55 to 0.83 THz) beam steering with a deflection angle from 59.09° to 34.88° at a modulation speed of 3 kHz (Figure 3h).

#### 2.1.3. High-Electron Mobility Transistor (HEMT)

**Figure 4:**
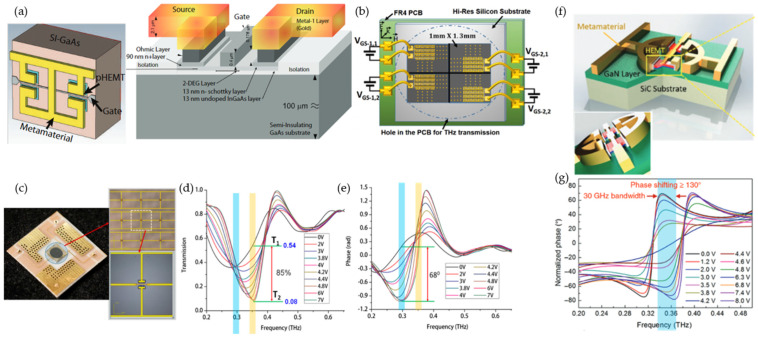
High-electron-mobility-transistor (HEMT)-enabled reconfigurable metasurface. (**a**,**b**) An HEMT-embedded metamaterial modulator with a speed of 10 MHz. (**a**) A simulation unit-cell model with HEMT beneath each split gap. The cross-sectional view is shown on the right. Reprinted from Ref. [61]. (**b**) A 2 × 2 spatial light modulator with a modulation depth of 33% at 0.46 THz. Reprinted from Ref. [62]. (**c**–**e**) Metasurface with 1 GHz modulation speed combining a dipolar array with a double-channel heterostructure. (**c**) Image of the fabricated metasurface. (**d**) Depth modulation of 85%. (**e**) Phase modulation of 68°. Reprinted from Ref. [66]. (**f**,**g**) Large phase modulator with HEMT embedded and an enhanced-resonance metasurface. (**f**) Schematic of the unit-cell structure. (**g**) Phase modulation of more than 130°. Reprinted from Ref. [67].

The instability of two-dimensional electron gases (2DEGs) in short-channel high-electron mobility transistors (HEMTs) leads to a resonant response at the geometrical plasmon frequency, which depends on the size and shape of the channel [59,60,61]. A pseudomorphic HEMT-integrated metadevice was first introduced in 2011 by D. Shrekenhamer et al. (Figure 4a) [61]. HEMTs were integrated beneath the capacitive gaps of a square electric-LC (ELC) resonator. The resonance response was reconfigured by changing the channel-carrier density through external bias voltage (1 V). The metadevice was fabricated using a commercial GaAs process and realized a modulation depth of 33% at 0.46 THz with a rate of 10 MHz. In 2016, the authors adopted the same meta-atom to demonstrate a 2 × 2 spatial light modulator (Figure 4b) [62].

Various structures have been designed to explore 2DEGs in HEMTs [63,64,65]. One such embedded HEMT with metal–insulator–metal (MIM) capacitors for amplitude modulation (45%) was introduced in [63] through a biasing voltage of 3 V at 0.58 THz. Reconfigurability with both amplitude and phase modulation is desirable. Double-channel heterostructures with two split channels of decreased polarized-carrier concentration were designed to support a nanoscale 2DEG layer with high concentration and mobility [66] (Figure 4c). An equivalent collective dipolar array was combined with a double-channel heterostructure. An external electrical signal was applied to control the electron concentration in the 10 nm thick 2DEG layers, which led to a resonant mode conversion between two dipolar resonances, providing fast amplitude and phase modulations. Depletion of the 2DEG layer shifted the dipolar resonance from the long central wire to the short one, resulting in a blueshift of the resonance frequency. This design demonstrated 1 GHz modulation speed for the first time and achieved 85% modulation depth (Figure 4d) and 68° phase shift (Figure 4e) at ~0.351 THz. By combining inductance–capacitance resonance and dipole resonance, an enhanced-resonance active HEMT metasurface was designed (Figure 4f), realizing a phase modulation of 137° at 0.35 THz with a biasing voltage of 8 V (Figure 4g) [67].

#### 2.1.4. Graphene

Graphene is a two-dimensional (2D) material of honeycomb structures formed by single-layer carbon atoms arranged in hexagonal lattices. Graphene has complex conductivity that supports the propagation of plasmonic modes at THz frequencies [68]. The surface conductivity can be efficiently controlled through a perpendicular-bias electric field that induces charge carriers to shift the graphene chemical potential (Fermi level) away from the Dirac point. Compared with conventional semiconductors, graphene has the attractive advantages of high electron mobility, considerable thermal conductivity, and strong mechanical ductility [69]. Hence, graphene has considerable potential to be applied in the THz frequency for dynamic wave control.

**Figure 5:**
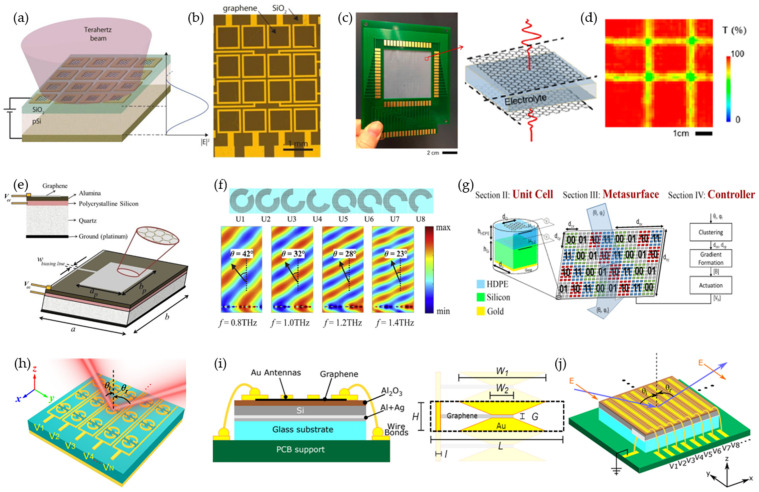
Graphene-based reconfigurable metasurface. (**a**,**b**) A 4 × 4 reflection modulator. (**a**) A schematic of the graphene-${\mathrm{SiO}}_{2}$-Si structures. The substrate has an optical thickness of an odd quarter wavelength. (**b**) Optical image of the graphene-enabled reflection modulator. Reprinted from Ref. [71]. (**c**,**d**) A 256-pixel spatial light modulator. (**c**) Photo of the modulator (left). The enlargement (right) shows the graphene−electrolyte–graphene unit-cell structure. (**d**) A THz transmission image at 0.1 THz with two rows and columns biased at +1.0 and −1.0 V, respectively. Reprinted from Ref. [72]. (**e**) Unit cell consisting of a square graphene patch for a tunable reflective metasurface at 1.3 THz. The cell has dimensions of a = b = 14 µm, $a_{p}\;$ =$\;b_{p}\;$ = 10 μm. Reproduced with permission from [68]. (**f**) Beam steering with graphene patterned in SRRs. Reprinted from Ref. [73]. (**g**) Digital metasurface using a graphene–insulator–graphene stack for beam steering. Reprinted from Ref. [81]. (**h**) Column-level controlled beam steering with graphene embedded with SRR structures. Reprinted from Ref. [82]. (**i**–**j**) Experimentally demonstrated beam-steering metasurface with graphene embedded with a bowtie structure. (**i**) Cross section of the experimentally demonstrated beam-steering metasurface (left) and its unit-cell structure (right). (**j**) Schematic of the metasurface with the individually biased column. Reprinted from Ref. [83].

A graphene-based electroabsorption modulator was demonstrated by placing atomically thin graphene layer on top of a dielectric substrate with a reflective metal back gate (Figure 5a) [70,71]. The substrate thickness was designed to be an odd multiple of a quarter-wavelength of the incident wave to enhance the modulation depth. With a biasing voltage of −10 V, the Fermi level in graphene was tuned at the Dirac point, realizing the maximal reflectance. The carrier concentration increased with increased voltage, resulting in enhanced absorption. The graphene layer was patterned using $O_{2}$ plasma to demonstrate a 4 × 4 pixelated reflectance modulator (Figure 5b) [71]. An alternative approach using electrolyte gating, producing higher charge densities to build a spatial phase modulator, was presented in [72] (Figure 5c). Large electric fields were generated at the graphene–electrolyte interface, giving rise to charge accumulation over a large area without an electrical short circuit and removing the thickness control. In order to build the 16 × 16 pixelated modulator, graphene was grown by chemical-vapor deposition (CVD) on a large area. Selective control of a single pixel was achieved by voltage biasing through the corresponding column and row (Figure 5d).

A reflect array combined with graphene for dynamic beam steering was first proposed using a square graphene patch (Figure 5e) [68]. Due to the slow wave propagation in the plasmonic mode of graphene, a patch was designed with much smaller dimensions (λ/10, compared with conventional conductors of λ/2) for a resonance response. This reduced interelement spacing allows for more efficient wavefront manipulation. By tuning the chemical potential from 0 to 0.52 eV, a phase coverage of 300° was obtained at 1.3 THz with a fixed patch dimension. To have full 360° phase coverage for dynamic-gradient phase control, graphene was designed with resonant structures (Figure 5f) [73,74] or combined with resonant metallic patterns [75,76]. A graphene-based coding metasurface for beam splitting was proposed by controlling the Fermi level (Figure 5g) [77,78,79,80,81]. Such coding metasurfaces also have great potential to be applied to security systems for message transmission [81]. Spatially selective column-level tuning was presented with graphene embedded in SRR, resulting in four deflection angles (5°, 11°, 17°, and 23°) at 1.05 THz (Figure 5h) [82]. Such graphene-based beam steering metasurfaces had only been tested in simulations. The first experimentally demonstrated design was illustrated in [83] (Figure 5i). The graphene strip was placed at the gap of a bowtie structure for field concentration. Each column was individually controlled, resulting in a maximum beam steering of ±25° at 1 THz (Figure 5j).

### 2.2. Optical Approaches

**Figure 6:**
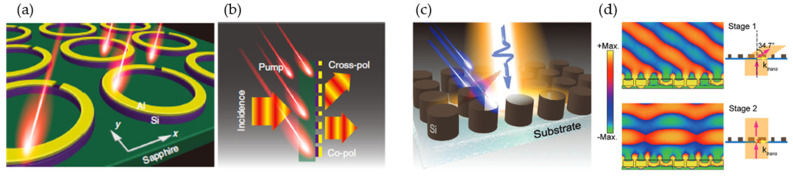
Semiconductor materials for temporal modulation. (**a**,**b**) Optical active polarization switching and dynamic beam splitting. (**a**) An illustration of the hybrid circular split-ring resonator (h-SRR) pumped by near-infrared femtosecond pulses. (**b**) An active polarizing beam splitter. Reprinted from Ref. [84]. (**c**,**d**) Spatiotemporal dielectric metasurfaces for beam steering. (**c**) Ultrafast femtosecond laser pulses (@ 800 nm, 100 fs) pump high-resistivity silicon on a quartz substrate, providing transient photocarriers for temporal modulation. (**d**) Temporal beam steering of 34.7° at 0.586 THz. Reprinted from Ref. [85].

In photosensitive semiconductors (e.g., silicon, GaAs, and conducting oxide), conductivity can be controlled by pumping carriers from the valence band to the conduction band using an external laser beam with photon energy higher than that of the bandgap [84]. This dynamic photoconductivity provides temporal modulation of the metasurface. Figure 6a shows a hybrid circular SRR with an aluminum SRR placed on a circular silicon ring [84]. The top layer SRR was designed with eight patterns for 360° phase coverage, realizing a wideband (0.6–1 THz) cross-polarized wavefront deflection from 51° to 28° (Figure 6b). Stimulating the silicon with an external optical laser pump (800 nm) increased its conductivity and closed the SRR gap, eliminating the beam-splitting and deflection effect. A similar concept was applied to high-resistivity silicon resonators (Figure 6c) [85]. A supercell consisting of four resonators was designed, realizing a beam-deflection angle of 34.7° at 0.586 THz with 5 mW laser-pump power (Figure 6d). The rise time for the transient photocarrier was 14 ps. This symmetry-preserved Huygens’ metasurface design achieved a high transmission efficiency of 90%.

### 2.3. Phase-Change Materials

Phase-change materials (PCMs) transform from the amorphous to the crystalline state or between different crystalline phases upon external stimulation [86]. Their reversible phase transition, thermal stability, and fast switching speed make PCMs extremely popular [87]. Various phase-change materials, such as vanadium dioxide (${\mathrm{VO}}_{2}$), chalcogenide phase-change materials, and liquid crystals (LCs), have been explored for reconfigurable metasurfaces.

#### 2.3.1. Vanadium Dioxide (${\mathrm{VO}}_{2}$)

**Figure 7:**
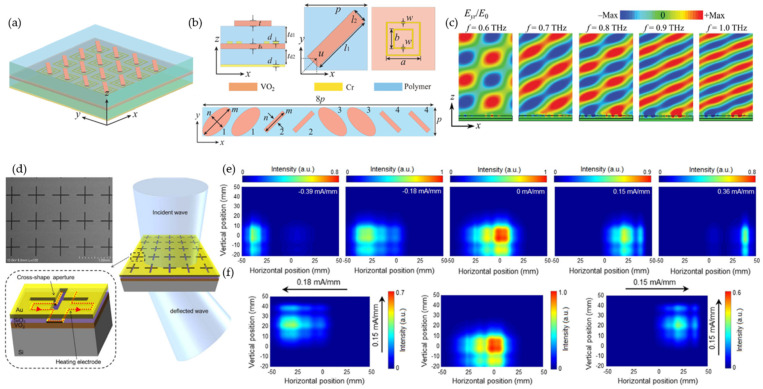
Phase-change materials of a vanadium-dioxide (${\mathrm{VO}}_{2}$)-enabled reconfigurable metasurface. (**a**–**c**) A thermally controlled reconfigurable metasurface with broadband absorption-to-reflection conversion. Reprinted from Ref. [90]. (**a**) Schematic of the ${\mathrm{VO}}_{2}$ integrated metasurface. (**b**) Unit-cell structure (top) and a unit-cell array for 2𝜋 phase control (bottom). (**c**) Broadband reflection when ${\mathrm{VO}}_{2}$ is in its fully metallic state. (**d**–**f**) An electronically controlled beam-steering metasurface operates at 0.1 THz. Reprinted from Ref. [89]. (**d**) Top-view scanning electron microscopic image of the metasurface (top left), unit-cell structure (bottom left), and beam steering (right). (**e**) Horizontal beam steering at −22°, −14°, 0°, 12°, and 22°. (**f**) Vertical beam steering at −14°, 0°, and 12°.

Vanadium dioxide (${\mathrm{VO}}_{2}$) has a unique atomic rearrangement of monoclinic phases, exhibiting semiconductor behavior at room temperature and rutile phases at high temperatures (~68°), experiencing an abrupt transition from insulator to metal when subjected to thermal, electrical, optical, or mechanical stimulation [88,89,90,91,92]. This insulator-to-metal transition property provided a diversified application for switching a metasurface between the broadband-absorber and half-wave plate states (Figure 7a) [90]. The metasurface had an absorption efficiency >90% at room temperature but became a reflector as the temperature rose above the phase-change temperature [90]. Supercells consisting of eight resonators were designed to have a beam-steering effect in the metallic state (Figure 7b). The metasurface had a simulated wideband deflection of ~28° at 0.8 THz (Figure 7c). Embedded ${\mathrm{VO}}_{2}$ with resonant structures for a multifunctional coding metasurface was also presented numerically [91,93,94,95]. An experimental demonstration of ${\mathrm{VO}}_{2}$-based reconfigurable beam steering was presented in [89] (Figure 7d). The unit cell had a cross-shaped aperture with a resistive heater electrode placed beneath for independent Joule-heating actuation. A 100 nm thick ${\mathrm{SiO}}_{2}$ layer was used to insulate the resistive-heater electrodes from the top Au layer. The refractive index of the ${\mathrm{VO}}_{2}$ was varied through temperature changes. The metasurface achieved a 44° beam deflection in both horizontal and vertical directions at 0.1 THz (Figure 7e,f). Recently, an electrically triggered ${\mathrm{VO}}_{2}$-based reconfigurable metasurface for both phase (90°) and amplitude (71% at 0.79 THz) modulation was experimentally demonstrated in [96]. And a multi-state 8 × 8 pixelated reflective modulator was demonstrated with a modulation speed of 1 kHz [97].

#### 2.3.2. Chalcogenide Phase-Change Materials

**Figure 8:**
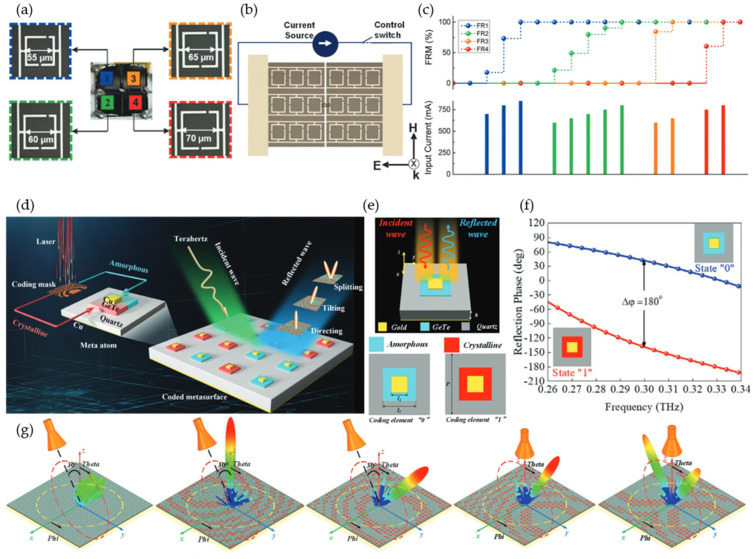
Chalcogenide phase-change materials enabled a reconfigurable metasurface. (**a**–**c**) Germanium–antimony–tellurium (GST) incorporated with Fano-resonance mate atoms for multicolor spatial light modulation. (**a**) A 2 × 2 array for four-color spatial light modulation. (**b**) Schematic of current biasing. (**c**) Multilevel Fano-resonance modulation (FRM) results from different input currents (stimulus period ≈15 s). Reprinted from Ref. [98]. (**d**–**g**) Phase-change GeTe material applied for a multifunctional coding metasurface. (**d**) Illustration of the coding metasurface. (**e**) GeTe- and gold-integrated unit cell with amorphous (insulating) state of GeTe and crystalline (conductive) state. (**f**) Reflected phase of 180° at 0.3 THz for the coding element at two different states. (**g**) Multifunctionality (beam tilting, directing, and splitting) is realized through different coding masks. Reprinted from Ref. [101].

Phase transitions of chalcogenide phase-change materials are mediated by nucleation dynamics, providing an analog response by continuously varying the crystallinity fraction. Moreover, these analog states are nonvolatile, requiring zero-hold power [86]. A chalcogenide phase-change material, germanium–antimony–tellurium (GST), a ternary compound made of germanium (Ge), antimony (Sb), and tellurium (Te), exhibits a reconfigurable phase-transition response between amorphous and crystalline states under external optical, electrical, or thermal stimulation. A multifunctional tunable metadevice was described in [98] using ${\mathrm{Ge}}_{2}{\mathrm{Sb}}_{2}{\mathrm{Te}}_{5}$. A dual-split asymmetric SRR was designed for Fano resonance (Figure 8a). Four meta-atoms designed for different THz frequency responses were configured in a 2 × 2 array. A spatially selective reconfiguration was achieved by Joule heating from the isolated biasing current (Figure 8b). Continuously increasing the material temperature led to increased terahertz conductivity and refractive index, providing multilevel resonance modulation states (reaching 100% at 850 mA) (Figure 8c).

Germanium telluride (GeTe) with a crystallization temperature of ~200 ℃ and a resistivity reduction of six orders has also been extensively studied for the construction of tunable metasurfaces [99,100]. Optical stimulus provides faster material phase-change modulation [98,101]. Recently, a two-bit coding metasurface based on GeTe was experimentally demonstrated with multifunctionality, including beam tilting, directing, and splitting at 0.3 THz (Figure 8d) [101]. A laser pulse excited the meta-atom between crystalline (conductive) and amorphous (insulating) states (Figure 8e), achieving a reflected phase difference of 180° (Figure 8f). Five different beam controls were demonstrated with five coding masks, as shown in Figure 8g.

#### 2.3.3. Liquid Crystals

**Figure 9:**
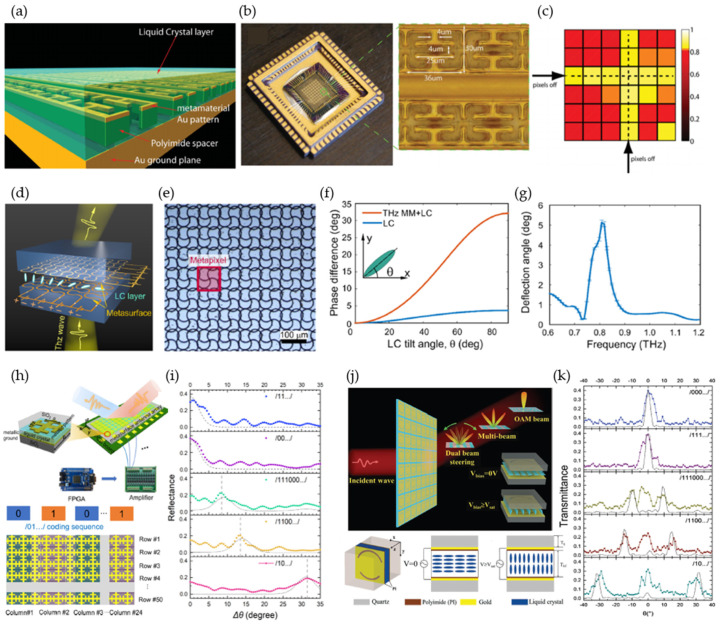
Liquid-crystal-enabled reconfigurable metasurface. (**a**–**c**) A spatial light modulator based on liquid crystals. (**a**) Schematic of metamaterial absorbers covered with a layer of liquid crystals. (**b**) Spatial light modulator device and an enlargement for the meta-atom dimensions. (**c**) A 6 × 6 pixelated absorption map measured at 3.725 THz. Reprinted from Ref. [102]. (**d**–**g**) A spatial phase modulator operating at 0.8 THz. (**d**) Schematic of the metasurface. (**e**) An optical microscopic image of the fabricated metasurface. (**f**) Phase difference as a function of liquid-crystal tilt angle. (**g**) Calculated beam deflection. Reprinted from Ref. [104]. (**h**,**i**) Programmable metasurface for beam steering. (**h**) Schematic of the beam steering metasurface with the control element (top). The unit cell consists of a liquid-crystal layer embedded between two metallic layers. Schematic of the metasurface with the applied coding sequence of /01.../(bottom). (**i**) Reflected angles for five different coding sequences with an incident angle of 20° at 0.672 THz. Reprinted from Ref. [105]. (**j**,**k**) Liquid crystal-based multifunctional transmissive coding metasurface. (**j**) Schematic of the functional metasurface (top) and the asymmetric unit-cell design (bottom). (**k**) Measured transmitted pattern for different coding sequences. Reprinted from Ref. [106].

Liquid crystals are attractive for their inherent birefringent properties, which depend on the orientation of liquid crystal molecules and can be effectively controlled by an external electric field or light [102,103,104,105]. Figure 9a shows a THz spatial light modulator based on liquid crystals combined with metamaterial absorbers [102]. The authors used an isothiocyanate-based liquid-crystal mixture to fill the space around the electric ring resonator (ERR). The spatial light modulator consisted of a 6 × 6 pixel array (Figure 9b), and each pixel was individually controlled by a 15 V peak-to-peak square waveform at 1 kHz. The applied electric field forced the liquid-crystal molecule to align with its direction, achieving 75% reflectivity modulation at 3.67 THz (Figure 9c).

The reconfigurable effective refractive index of liquid crystal makes it suitable for both amplitude and phase modulation [103]. Figure 9d shows a spatial phase modulator of a nematic liquid-crystal layer sandwiched between two orthogonally placed metasurfaces [104]. Meandering wires enabled the electric potential of each pixel to be selectively and spatially addressed. The anisotropic metapixel (unit cell) consisted of two metallic split rings to enhance liquid-crystal birefringence (Figure 9e,f). The maximum phase change (32°) resulted from a 90° tilt angle. A biasing voltage of 20 V achieved a deflection angle of 5° at 0.8 THz (Figure 9g). Combining liquid crystals with a programmable metasurface enabled more advanced dynamic beam steering (Figure 9h) [105]. A 25 μm thick liquid-crystal layer was embedded inside a metal–insulator–metal (MIM) resonator with a top metal layer patterned in the Jerusalem cross structure. Bias voltages of 0 and 40 V were applied to have “0” and “1” states with 180° phase differences while maintaining the same reflection amplitude. By changing the phase gradient through coding pattern, different reflected angles were achieved at 0.672 THz, for an incident angle of 20°, as shown in Figure 9i. The maximum acquired deflection angle was 32°, with a reflection efficiency of 19.1%. By designing unit cells with more phase changes, this method can be further extended to two-bit- or three-bit-coding liquid-crystal metasurfaces, achieving a wider beam-deflection angle and higher reflection efficiency. Recently, an liquid crystal -based transmissive coding metasurface was demonstrated for multifunctional control (Figure 9j) [106]. An asymmetric metasurface pattern was designed for Fano resonance, realizing a transmission efficiency as high as ~50% at 0.426 THz. Figure 9k shows the measured beam-splitting patterns using different coding sequences.

### 2.4. Micro-Electromechanical-System (MEMS)

**Figure 10:**
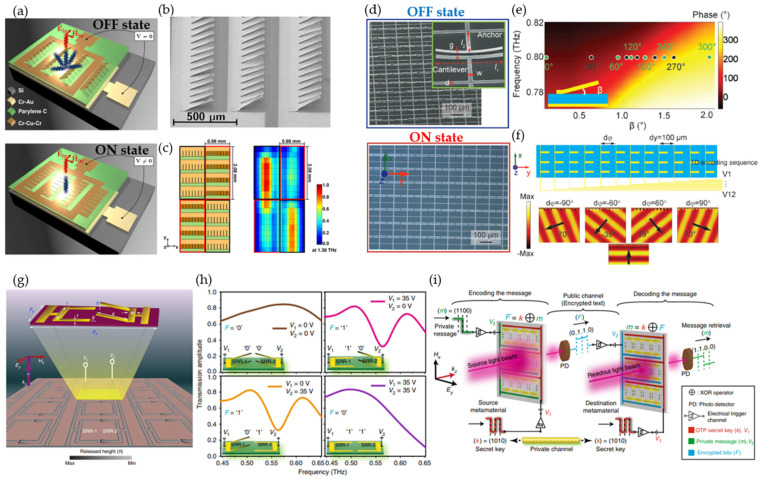
MEMS-enabled reconfigurable metasurface. (**a**–**c**) Micromirror array for the wideband spatial light modulator. (**a**) Schematic of a single-pixel in OFF state (top) and ON-state (bottom) for a bias voltage of 0 V and 37 V, respectively. (**b**) SEM image of the inclined mirrors. (**c**) Model of a 2 × 2-pixel SLM with two ON pixels (highlighted by black frames) along one diagonal (left) and its corresponding measured-intensity distribution (right) at 1.38 THz. Reprinted from Ref. [108]. (**d**–**f**) MEMS-based metal–insulator–metal metadevices for beam steering. (**d**) Images of the fabricated metasurface in “ON” and “OFF” states. (**e**) Simulated phase response as a function of the cantilever angles. (**f**) Simulated dynamic beam steering with six-digit control. Reprinted from Ref. [109]. (**g**–**i**) Reconfigurable MEMS Fano metasurfaces for logic operations in cryptographic wireless communication networks. (**g**) Unit-cell model of the metasurface. (**h**) Measured far-field transmission spectra showing the exclusive-OR (XOR) logic feature for various voltage states of the SRRs at 0.56 THz. (**i**) Implementation of the XOR logic for OTP-secured wireless communication channels. Reprinted from Ref. [110].

In contrast to other tuning mechanisms that alter the properties of materials, micro-electromechanical-system (MEMS) metasurfaces directly change the structural geometry of the unit cell, transforming the electromagnetic wave responses. Moreover, advanced and developed MEMS manufacturing makes it attractive for reconfigurable THz devices [107]. The simplest microstructure cantilever has been extensively studied and was embedded in a metasurface for active tuning [44]. A wideband spatial light modulator was built using an array of 768 actuatable mirrors, with a length of 220 μm and a width of 100 μm (Figure 10a) [108]. These dimensions were selected to reduce diffraction from individual mirrors and to increase the pixel-to-pixel modulation contrast of the spatial light modulator. A cantilever consisting of chrome –copper–chrome multilayers had intrinsic residual stress forcing it to tilt up with an angle of 35° (Figure 10b), which minimized back-diffracted waves in the incident direction. The mirrors were pulled down to the substrate by applying a bias voltage of 37 V. The SLM was built with micromirror arrays based on the grating concept to have a wide operational spectral range. The authors designed a spatial light modulator with 4 × 6 independently switchable pixels. Each pixel consisted of 4 × 8 micromirrors with a pixel size of 1 mm × 2 mm (Figure 10c). The modulation contrast was higher than 50% over the frequency range from 0.97 THz to 2.28 THz, with a peak modulation contrast of 87% at 1.38 THz. This method allows for almost arbitrary spatial pixel sizes by collectively switching the corresponding group of mirrors.

Beam steering was demonstrated by a cantilever designed for electrical LC resonance (Figure 10d) [109]. By controlling the suspension angle (from 2° to 0°) of the bimorph cantilever through biasing voltage (from 0 V to 30 V), a phase coverage of 310° was achieved at 0.8 THz (Figure 10e). Steering angles of ±70° and ±39° were demonstrated through simulation by grouping twelve columns as a super cell and controlling each column individually (Figure 10f). Each unit cell could potentially be addressed separately to have unit-cell-level control to enable a programmable MEMS metasurface. A MEMS-based metasurface for wireless security encoding was demonstrated using square double SRRs, forming a Fano resonator (Figure 10g) [110]. Independent control of the two SRRs provided four transmission states for an exclusive-OR (XOR) logic operation (Figure 10h). An application performing the XOR logic operation for one-time-pad (OTP) security in wireless transmission is illustrated in Figure 10i. A private message (m) was encoded with a secret key (k) using the XOR logic operation before sending it out for public-channel transmission, and the original message was decrypted at the destination through the inverse XOR operation. This fast encryption method could be extended to other wireless communication networks requiring high security.

## 3. Discussion

THz RISs can dynamically modify the wave propagation direction, thereby enhancing wireless network efficiency, are crucial for actualization of 6G communication links. Reconfigurable metasurfaces with the function of pixel-level amplitude modulation and tunable beam steering are summarized in Table 1 and Table 2, respectively, according to tuning elements. Electrical control allows for more precise, spatially selective pixel-level modulation but requires additional wires and complex control circuits. Optical control usually modulates the whole surface with a laser pump, but localization may be possible through an additional coding mask [101]. Tuning elements can be combined to add additional reconfigurable freedom [111,112]. Other reconfigurable methods, such as mechanical [113] and microfluid-based [114] tuning, are also feasible. RISs realized through industry-standard fabrication processes have greater potential for large-scale construction, which is required for future 6G smart cities. Moreover, emerging 2D van der Waals materials using surface-plasmon polaritons provide new approaches for dynamic tunning in the THz spectrum [115,116,117,118,119,120]. Graphene plasmons demonstrate lower loss than conventional metal-based plasmonic metasurfaces [121,122]. Recently discovered phonon polaritons in van der Waals crystals exhibit remarkably low losses [123,124,125]. These phonon polaritons can be tunned through chemical intercalation [126,127,128], twisted stacking [129], and heterostructures [130,131,132,133].

Since the emergence of multiple-input, multiple-output (MIMO) technology for wireless communication, polarization modulation (PoM) has gained more and more attention thanks to its excellent signal distinguishability, thereby increasing spectrum efficiency [134,135]. Metasurfaces with active elements for polarization modulation at THz frequencies were investigated in [136,137,138,139] and were found to have great potential to be utilized for future 6G wireless communications. Another promising area for future THz RIS applications is space-time-coding digital metasurfaces with have multifrequency control for beam shaping and steering [140]. Cell-free massive MIMO for mobile access is expected in 6G based on massive MIMO in 5G. Hybrid RISs with MIMO constitute an important future research direction.

## Figures and Tables

**Table 1:** Pixel-level amplitude-modulation performance comparison of various tuning elements for THz RIS.

Tuning Element	Tuning Mechanism	Control Method	Frequency (THz)	Array Size	No. of State/Pixel	Modulation Depth	Modulation Speed	Power Consumption	Ref.
CMOS transistor	Field-effect-transistor (FET)-based switch	Bias voltage	0.3	12 × 12	256	25 dB	5 GHz	~mW (1.2 V)	Exp. [52]
Schottky diode	Depletion of substrate charge carrier	Bias voltage	0.36	4 × 4	2	40%	3 kHz	14 V	Exp. [55]
		Bias voltage	2.72, 3.27, 3.81, 4.34	8 × 8	2	62%	12 MHz	−26.5 V	Exp. [56]
HEMT	Depletion of channel-carrier density	Bias voltage	0.45	2 × 2	2	36%	10 MHz	<1 mW (1 V)	Exp. [62]
Graphene	Fermi level	Bias voltage	0.59	4 × 4	2	~50 to ~30%	6 kHz	−10 V	Exp. [71]
		Bias voltage	0.1	16 × 16	2	80%	1 kHz	4 V	Exp. [72]
GST	Joule heating	Bias current	0.69, 0.64, 0.6, 0.56	2 × 2	8	100%	15 s	850 mA	Exp. [98]
Liquid crystal	Birefringence effect	Bias voltage	3.670	6 × 6	2	75%	1 kHz	15 V	Exp. [102]
MEMS	Structural deformation	Bias voltage	0.97 to 2.28	4 × 6	2	50%	1 kHz	37 V	Exp. [108]

**Table 2:** Beam-steering performance comparison of various tuning elements for THz RIS.

Tuning Element	Tuning Mechanism	Control Method	Frequency (THz)	Deflection Angle	Modulation Speed	Power Consumption	Ref.
CMOS transistor	Field-effect-transistor (FET)-based switch	Bias voltage	0.3	±30°	5 GHz	240 µW (1.2 V)	Exp. [52]
Schottky diode	Depletion of substrate charge carrier	Bias voltage	0.4	36.1°	1 kHz	−13 V	Exp. [57]
		Bias voltage	0.55–0.83	59.09°–34.88°	3 kHz	−10 V	Exp. [58]
Graphene	Fermi level	Bias voltage	0.8–1.4	42°–23°	ps	--	Sim. [73]
		Bias voltage	1.05	5°, 11°, 17°, 23°	ps	--	Sim. [82]
		Bias voltage	1	−5°,10°,17.5°,	60 GHz	26 V, −44 V	Exp. [83]
Silicon	Photoconductivity	Laser pulses	0.6–1	51°–28°	30 ps	1.9 mJ/${\mathrm{cm}}^{2}$	Exp. [84]
		Laser pulses	0.586	±34.7°	14 ps	5.0 mW	Exp. [85]
${\mathrm{VO}}_{2}$	Phase change	Joule heating	0.7–1	±28°	<1 kHz	--	Sim. [90]
		Joule heating	0.1	±22°, −14°, 12°	<1 kHz	10 mV	Exp. [89]
GeTe	Phase change	Laser pulses	0.3	±30°	35 ns	190 mJ/$cm^{2}$	Exp. [101]
Liquid crystals	Birefringence effect	Bias voltage	0.8	±4.5°	75 ms	20 V	Exp. [104]
		Bias voltage	0.672	8.5°, 13.5°, 31.5°	100 Hz	40 V	Exp. [105]
		Bias voltage	0.426	−9°,−15°,−29°, 9°, 16°,30°	1 kHz	10 V	Exp. [106]
MEMS	Structural deformation	Bias voltage	0.8	±39°, ±70°	sub-MHz	20 V	Sim. [109]

## References

[1] Yang P., Xiao Y., Xiao M., Li S. (2019). 6G Wireless Communications: Vision and Potential Techniques. IEEE Netw..

[2] Giordani M., Polese M., Mezzavilla M., Rangan S., Zorzi M. (2020). Toward 6G Networks: Use Cases and Technologies. IEEE Commun. Mag..

[3] Dang S., Amin O., Shihada B., Alouini M.S. (2020). What Should 6G Be?. Nat. Electron..

[4] Imoize A.L., Adedeji O., Tandiya N., Shetty S. (2021). 6G Enabled Smart Infrastructure for Sustainable Society: Opportunities, Challenges, and Research Roadmap. Sensors.

[5] Akyildiz I.F., Kak A., Nie S. (2020). 6G and Beyond: The Future of Wireless Communications Systems. IEEE Access.

[6] Chowdhury M.Z., Shahjalal M., Ahmed S., Jang Y.M. (2020). 6G Wireless Communication Systems: Applications, Requirements, Technologies, Challenges, and Research Directions. IEEE Open J. Commun. Soc..

[7] Kleine-Ostmann T., Nagatsuma T. (2011). A Review on Terahertz Communications Research. J. Infrared Millim. Terahertz Waves.

[8] Elayan H., Amin O., Shihada B., Shubair R.M., Alouini M.-S. (2019). Terahertz Band: The Last Piece of RF Spectrum Puzzle for Communication Systems. IEEE Open J. Commun. Soc..

[9] O’Hara J.F., Ekin S., Choi W., Song I. (2019). A Perspective on Terahertz Next-Generation Wireless Communications. Technologies.

[10] Jamshed M.A., Nauman A., Abbasi M.A.B., Kim S.W. (2020). Antenna Selection and Designing for THz Applications: Suitability and Performance Evaluation: A Survey. IEEE Access.

[11] Peng B., Guan K., Rey S., Kurner T. (2019). Power-Angular Spectra Correlation Based Two Step Angle of Arrival Estimation for Future Indoor Terahertz Communications. IEEE Trans. Antennas Propag..

[12] Liaskos C., Nie S., Tsioliaridou A., Pitsillides A., Ioannidis S., Akyildiz I. (2018). A New Wireless Communication Paradigm through Software-Controlled Metasurfaces. IEEE Commun. Mag..

[13] Huang C., Zappone A., Alexandropoulos G.C., Debbah M., Yuen C. (2019). Reconfigurable Intelligent Surfaces for Energy Efficiency in Wireless Communication. Proceedings of the IEEE Transactions on Wireless Communications.

[14] Basar E., Renzo M.D., de Rosny J., Debbah M., Alouini M.-S., Zhang R., di Renzo M. (2019). Wireless Communications Through Reconfigurable Intelligent Surfaces. IEEE Access.

[15] Elmossallamy M.A., Zhang H., Song L., Seddik K.G., Han Z., Li G.Y. (2020). Reconfigurable Intelligent Surfaces for Wireless Communications: Principles, Challenges, and Opportunities. IEEE Trans. Cogn. Commun. Netw..

[16] Alexandropoulos G.C., Lerosey G., Debbah M., Fink M. (2020). Reconfigurable Intelligent Surfaces and Metamaterials: The Potential of Wave Propagation Control for 6G Wireless Communications. arXiv.

[17] Di B., Zhang H., Song L., Li Y., Han Z., Poor H.V. (2020). Hybrid Beamforming for Reconfigurable Intelligent Surface Based Multi-User Communications: Achievable Rates with Limited Discrete Phase Shifts. IEEE J. Sel. Areas Commun..

[18] Yang B., Cao X., Huang C., Guan Y.L., Yuen C., di Renzo M., Niyato D., Debbah M., Hanzo L. (2021). Spectrum Learning-Aided Reconfigurable Intelligent Surfaces for “Green” 6G Networks. IEEE Netw..

[19] Liaskos C., Tsioliaridou A., Pitsillides A., Akyildiz I.F., Kantartzis N.V., Lalas A.X., Dimitropoulos X., Ioannidis S., Kafesaki M., Soukoulis C.M. (2015). Design and Development of Software Defined Metamaterials for Nanonetworks. IEEE Circuits Syst. Mag..

[20] Abadal S., Liaskos C., Tsioliaridou A., Ioannidis S., Pitsillides A., Sole-Pareta J., Alarcon E., Cabellos-Aparicio A. (2017). Computing and Communications for the Software-Defined Metamaterial Paradigm: A Context Analysis. IEEE Access.

[21] Zhao J. (2019). A Survey of Intelligent Reflecting Surfaces (IRSs): Towards 6G Wireless Communication Networks. arXiv.

[22] Pillay N., Xu H. (2020). Large Intelligent Surfaces: Random Waypoint Mobility and Two-Way Relaying. Int. J. Commun. Syst..

[23] Yu N., Capasso F. (2014). Flat Optics with Designer Metasurfaces. Nat. Mater..

[24] Yu N., Genevet P., Kats M.A., Aieta F., Tetienne J.-P., Capasso F., Gaburro Z. (2011). Light Propagation with Phase Discontinuities: Generalized Laws of Reflection and Refraction. Science.

[25] Wong J.P.S., Epstein A., Eleftheriades G.V. (2016). Reflectionless Wide-Angle Refracting Metasurfaces. IEEE Antennas Wirel. Propag. Lett..

[26] Chen M., Abdo-Sánchez E., Epstein A., Eleftheriades G.V. (2018). Theory, Design, and Experimental Verification of a Reflectionless Bianisotropic Huygens’ Metasurface for Wide-Angle Refraction. Phys. Rev. B.

[27] Ho J.S., Qiu B., Tanabe Y., Yeh A.J., Fan S., Poon A.S.Y. (2015). Planar Immersion Lens with Metasurfaces. Phys. Rev. B-Condens. Matter Mater. Phys..

[28] Zhuang Z.P., Chen R., Fan Z.B., Pang X.N., Dong J.W. (2019). High Focusing Efficiency in Subdiffraction Focusing Metalens. Nanophotonics.

[29] Yang F., Raeker B.O., Nguyen D.T., Miller J.D., Xiong Z., Grbic A., Ho J.S. (2020). Antireflection and Wavefront Manipulation with Cascaded Metasurfaces. Phys. Rev. Appl..

[30] Cui T.J., Qi M.Q., Wan X., Zhao J., Cheng Q. (2014). Coding Metamaterials, Digital Metamaterials and Programmable Metamaterials. Light Sci. Appl..

[31] della Giovampaola C., Engheta N. (2014). Digital Metamaterials. Nat. Mater..

[32] Gao L.H., Cheng Q., Yang J., Ma S.J., Zhao J., Liu S., Chen H.B., He Q., Jiang W.X., Ma H.F. (2015). Broadband Diffusion of Terahertz Waves by Multi-Bit Coding Metasurfaces. Light Sci. Appl..

[33] Liu S., Cui T.J., Zhang L., Xu Q., Wang Q., Wan X., Gu J.Q., Tang W.X., Qing Qi M., Han J.G. (2016). Convolution Operations on Coding Metasurface to Reach Flexible and Continuous Controls of Terahertz Beams. Adv. Sci..

[34] Ma Q., Shi C.B., Bai G.D., Chen T.Y., Noor A., Cui T.J. (2017). Beam-Editing Coding Metasurfaces Based on Polarization Bit and Orbital-Angular-Momentum-Mode Bit. Adv. Opt. Mater..

[35] Liu S., Cui T.J. (2017). Concepts, Working Principles, and Applications of Coding and Programmable Metamaterials. Adv. Opt. Mater..

[36] Ma Q., Chen L., Jing H.B., Hong Q.R., Cui H.Y., Liu Y., Li L., Cui T.J. (2019). Controllable and Programmable Nonreciprocity Based on Detachable Digital Coding Metasurface. Adv. Opt. Mater..

[37] Wu L.W., Ma H.F., Wu R.Y., Xiao Q., Gou Y., Wang M., Wang Z.X., Bao L., Wang H.L., Qing Y.M. (2020). Transmission-Reflection Controls and Polarization Controls of Electromagnetic Holograms by a Reconfigurable Anisotropic Digital Coding Metasurface. Adv. Opt. Mater..

[38] Wan X., Qi M.Q., Chen T.Y., Cui T.J. (2016). Field-Programmable Beam Reconfiguring Based on Digitally-Controlled Coding Metasurface. Sci. Rep..

[39] Yang H., Cao X., Yang F., Gao J., Xu S., Li M., Chen X., Zhao Y., Zheng Y., Li S. (2016). A Programmable Metasurface with Dynamic Polarization, Scattering and Focusing Control. Sci. Rep..

[40] Han R., Hu Z., Wang C., Holloway J., Yi X., Kim M., Mawdsley J. (2019). Filling the Gap: Silicon Terahertz Integrated Circuits Offer Our Best Bet. IEEE Microw. Mag..

[41] Abadal S., Cui T.J., Low T., Georgiou J. (2020). Programmable Metamaterials for Software-Defined Electromagnetic Control: Circuits, Systems, and Architectures. IEEE J. Emerg. Sel. Top. Circuits Syst..

[42] Bao L., Cui T.J. (2020). Tunable, Reconfigurable, and Programmable Metamaterials. Microw. Opt. Technol. Lett..

[43] Tsilipakos O., Tasolamprou A.C., Pitilakis A., Liu F., Wang X., Mirmoosa M.S., Tzarouchis D.C., Abadal S., Taghvaee H., Liaskos C. (2020). Toward Intelligent Metasurfaces: The Progress from Globally Tunable Metasurfaces to Software-Defined Metasurfaces with an Embedded Network of Controllers. Adv. Opt. Mater..

[44] Pitchappa P., Kumar A., Singh R., Lee C., Wang N. (2021). Terahertz MEMS Metadevices. J. Micromech. Microeng..

[45] Xu J., Yang R., Fan Y., Fu Q., Zhang F. (2021). A Review of Tunable Electromagnetic Metamaterials with Anisotropic Liquid Crystals. Front. Phys..

[46] Mandal A., Cui Y., McRae L., Gholipour B. (2021). Reconfigurable Chalcogenide Phase Change Metamaterials: A Material, Device, and Fabrication Perspective. J. Phys. Photonics.

[47] Guo T., Argyropoulos C. (2021). Recent Advances in Terahertz Photonic Technologies Based on Graphene and Their Applications. Adv. Photonics Res..

[48] Li L., Jun Cui T., Ji W., Liu S., Ding J., Wan X., Bo Li Y., Jiang M., Qiu C.W., Zhang S. (2017). Electromagnetic Reprogrammable Coding-Metasurface Holograms. Nat. Commun..

[49] Zhang X.G., Jiang W.X., Jiang H.L., Wang Q., Tian H.W., Bai L., Luo Z.J., Sun S., Luo Y., Qiu C.W. (2020). An Optically Driven Digital Metasurface for Programming Electromagnetic Functions. Nat. Electron..

[50] Huang C., Zhang C., Yang J., Sun B., Zhao B., Luo X. (2017). Reconfigurable Metasurface for Multifunctional Control of Electromagnetic Waves. Adv. Opt. Mater..

[51] Luo Z., Wang Q., Zhang X.G., Wu J.W., Dai J.Y., Zhang L., Wu H.T., Zhang H.C., Ma H.F., Cheng Q. (2019). Intensity-Dependent Metasurface with Digitally Reconfigurable Distribution of Nonlinearity. Adv. Opt. Mater..

[52] Venkatesh S., Lu X., Saeidi H., Sengupta K. (2020). A High-Speed Programmable and Scalable Terahertz Holographic Metasurface Based on Tiled CMOS Chips. Nat. Electron..

[53] Liu Y., Sun T., Xu Y., Wu X., Bai Z., Sun Y., Li H., Zhang H., Chen K., Ruan C. (2021). Active Tunable THz Metamaterial Array Implemented in CMOS Technology. J. Phys. D Appl. Phys..

[54] Chen H.T., Padilla W.J., Zide J.M.O., Gossard A.C., Taylor A.J., Averitt R.D. (2006). Active Terahertz Metamaterial Devices. Nature.

[55] Chan W.L., Chen H.T., Taylor A.J., Brener I., Cich M.J., Mittleman D.M. (2009). A Spatial Light Modulator for Terahertz Beams. Appl. Phys. Lett..

[56] Shrekenhamer D., Montoya J., Krishna S., Padilla W.J. (2013). Four-Color Metamaterial Absorber THz Spatial Light Modulator. Adv. Opt. Mater..

[57] Karl N., Reichel K., Chen H.T., Taylor A.J., Brener I., Benz A., Reno J.L., Mendis R., Mittleman D.M. (2014). An Electrically Driven Terahertz Metamaterial Diffractive Modulator with More than 20 DB of Dynamic Range. Appl. Phys. Lett..

[58] Su X., Ouyang C., Xu N., Cao W., Wei X., Song G., Gu J., Tian Z., O’Hara J.F., Han J. (2015). Active Metasurface Terahertz Deflector with Phase Discontinuities. Opt. Express.

[59] Dyakonov M., Shur M. (1993). Shallow Water Analogy for a Ballistic Field Effect Transistor: New Mechanism of Plasma Wave Generation by Dc Current. Phys. Rev. Lett..

[60] Dyakonov M., Shur M. (1996). Detection, Mixing, and Frequency Multiplication of Terahertz Radiation by Two-Dimensional Electronic Fluid. IEEE Trans. Electron Devices.

[61] Shrekenhamer D., Rout S., Strikwerda A.C., Bingham C., Averitt R.D., Sonkusale S., Padilla W.J. (2011). High Speed Terahertz Modulation from Metamaterials with Embedded High Electron Mobility Transistors. Opt. Express.

[62] Rout S., Sonkusale S.R. (2016). A Low-Voltage High-Speed Terahertz Spatial Light Modulator Using Active Metamaterial. APL Photonics.

[63] Nouman M.T., Kim H.W., Woo J.M., Hwang J.H., Kim D., Jang J.H. (2016). Terahertz Modulator Based on Metamaterials Integrated with Metal-Semiconductor-Metal Varactors. Sci. Rep..

[64] Zhao Y., Wang L., Zhang Y., Qiao S., Liang S., Zhou T., Zhang X., Guo X., Feng Z., Lan F. (2019). High-Speed Efficient Terahertz Modulation Based on Tunable Collective-Individual State Conversion within an Active 3 Nm Two-Dimensional Electron Gas Metasurface. Nano Lett..

[65] Lee G., Nouman M.T., Hwang J.H., Kim H.W., Jang J.H. (2018). Enhancing the Modulation Depth of a Dynamic Terahertz Metasurface by Integrating into an Asymmetric Fabry-Pérot Cavity. AIP Adv..

[66] Zhang Y., Qiao S., Liang S., Wu Z., Yang Z., Feng Z., Sun H., Zhou Y., Sun L., Chen Z. (2015). Gbps Terahertz External Modulator Based on a Composite Metamaterial with a Double-Channel Heterostructure. Nano Lett..

[67] Zhang Y., Zhao Y., Liang S., Zhang B., Wang L., Zhou T., Kou W., Lan F., Zeng H., Han J. (2018). Large Phase Modulation of THz Wave via an Enhanced Resonant Active HEMT Metasurface. Nanophotonics.

[68] Carrasco E., Tamagnone M., Perruisseau-Carrier J. (2013). Tunable Graphene Reflective Cells for THz Reflectarrays and Generalized Law of Reflection. Appl. Phys. Lett..

[69] Wang R., Ren X.G., Yan Z., Jiang L.J., Sha W.E.I., Shan G.C. (2019). Graphene Based Functional Devices: A Short Review. Front. Phys..

[70] Sensale-Rodriguez B., Yan R., Rafique S., Zhu M., Li W., Liang X., Gundlach D., Protasenko V., Kelly M.M., Jena D. (2012). Extraordinary Control of Terahertz Beam Reflectance in Graphene Electro-Absorption Modulators. Nano Lett..

[71] Sensale-Rodriguez B., Rafique S., Yan R., Zhu M., Protasenko V., Jena D., Liu L., Xing H.G. (2013). Terahertz Imaging Employing Graphene Modulator Arrays. Opt. Express.

[72] Malevich Y., Ergoktas M.S., Bakan G., Steiner P., Kocabas C. (2020). Video-Speed Graphene Modulator Arrays for Terahertz Imaging Applications. ACS Photonics.

[73] Chen D., Yang J., Huang J., Bai W., Zhang J., Zhang Z., Xu S., Xie W. (2019). The Novel Graphene Metasurfaces Based on Split-Ring Resonators for Tunable Polarization Switching and Beam Steering at Terahertz Frequencies. Carbon.

[74] Chen D., Yang J., Huang J., Zhang Z., Xie W., Jiang X., He X., Han Y., Zhang Z., Yu Y. (2020). Continuously Tunable Metasurfaces Controlled by Single Electrode Uniform Bias-Voltage Based on Nonuniform Periodic Rectangular Graphene Arrays. Opt. Express.

[75] Zhang Y., Feng Y., Zhao J., Jiang T., Zhu B. (2017). Terahertz Beam Switching by Electrical Control of Graphene-Enabled Tunable Metasurface. Sci. Rep..

[76] Xu J., Liu W., Song Z. (2021). Graphene-Based Terahertz Metamirror with Wavefront Reconfiguration. Opt. Express.

[77] Xiao B., Zhang Y., Tong S., Yu J., Xiao L. (2020). Novel Tunable Graphene-Encoded Metasurfaces on an Uneven Substrate for Beam-Steering in Far-Field at the Terahertz Frequencies. Opt. Express.

[78] Xu J., Liu W., Song Z. (2021). Terahertz Dynamic Beam Steering Based on Graphene Coding Metasurfaces. IEEE Photonics J..

[79] Momeni A., Rouhi K., Rajabalipanah H., Abdolali A. (2018). An Information Theory-Inspired Strategy for Design of Re-Programmable Encrypted Graphene-Based Coding Metasurfaces at Terahertz Frequencies. Sci. Rep..

[80] Hosseininejad S.E., Rouhi K., Neshat M., Faraji-Dana R., Cabellos-Aparicio A., Abadal S., Alarcón E. (2019). Reprogrammable Graphene-Based Metasurface Mirror with Adaptive Focal Point for THz Imaging. Sci. Rep..

[81] Hosseininejad S.E., Rouhi K., Neshat M., Cabellos-Aparicio A., Abadal S., Alarcon E. (2019). Digital Metasurface Based on Graphene: An Application to Beam Steering in Terahertz Plasmonic Antennas. IEEE Trans. Nanotechnol..

[82] Wang B., Luo X., Lu Y., Li G. (2021). Full 360° Terahertz Dynamic Phase Modulation Based on Doubly Resonant Graphene–Metal Hybrid Metasurfaces. Nanomaterials.

[83] Tamagnone M., Capdevila S., Lombardo A., Wu J., Centeno A., Zurutuza A., Ionescu A.M., Ferrari A.C., Mosig J.R. (2018). Graphene Reflectarray Metasurface for Terahertz Beam Steering and Phase Modulation. arXiv.

[84] Cong L., Srivastava Y.K., Zhang H., Zhang X., Han J., Singh R. (2018). All-Optical Active THz Metasurfaces for Ultrafast Polarization Switching and Dynamic Beam Splitting. Light Sci. Appl..

[85] Cong L., Singh R. (2020). Spatiotemporal Dielectric Metasurfaces for Unidirectional Propagation and Reconfigurable Steering of Terahertz Beams. Adv. Mater..

[86] Wuttig M., Bhaskaran H., Taubner T. (2017). Phase-Change Materials for Non-Volatile Photonic Applications. Nat. Photonics.

[87] Raeis-Hosseini N., Rho J. (2017). Metasurfaces Based on Phase-Change Material as a Reconfigurable Platform for Multifunctional Devices. Materials.

[88] Wang L., Zhang Y., Guo X., Chen T., Liang H., Hao X., Hou X., Kou W., Zhao Y., Zhou T. (2019). A Review of THz Modulators with Dynamic Tunable Metasurfaces. Nanomaterials.

[89] Hashemi M.R.M., Yang S.H., Wang T., Sepúlveda N., Jarrahi M. (2016). Electronically-Controlled Beam-Steering through Vanadium Dioxide Metasurfaces. Sci. Rep..

[90] Ding F., Zhong S., Bozhevolnyi S.I. (2018). Vanadium Dioxide Integrated Metasurfaces with Switchable Functionalities at Terahertz Frequencies. Adv. Opt. Mater..

[91] Li J., Yang Y., Li J., Zhang Y., Zhang Z., Zhao H., Li F., Tang T., Dai H., Yao J. (2020). All-Optical Switchable Vanadium Dioxide Integrated Coding Metasurfaces for Wavefront and Polarization Manipulation of Terahertz Beams. Adv. Theory Simul..

[92] Wang S., Kang L., Werner D.H. (2017). Hybrid Resonators and Highly Tunable Terahertz Metamaterials Enabled by Vanadium Dioxide (VO_2_). Sci. Rep..

[93] Wang H., Deng L., Zhang C., Qu M., Wang L., Li S. (2019). Dual-Band Reconfigurable Coding Metasurfaces Hybridized with Vanadium Dioxide for Wavefront Manipulation at Terahertz Frequencies. Microw. Opt. Technol. Lett..

[94] Pan W.-M., Li J.-S., Zhou C. (2021). Switchable Digital Metasurface Based on Phase Change Material in the Terahertz Region. Opt. Mater. Express.

[95] Shabanpour J., Beyraghi S., Cheldavi A. (2020). Ultrafast Reprogrammable Multifunctional Vanadium-Dioxide-Assisted Metasurface for Dynamic THz Wavefront Engineering. Sci. Rep..

[96] Jiang M., Hu F., Zhang L., Quan B., Xu W., Du H., Xie D., Chen Y. (2021). Electrically Triggered VO2 Reconfigurable Metasurface for Amplitude and Phase Modulation of Terahertz Wave. J. Lightwave Technol..

[97] Chen B., Wu J., Li W., Zhang C., Fan K., Xue Q., Chi Y., Wen Q., Jin B., Chen J. (2022). Programmable Terahertz Metamaterials with Non-Volatile Memory. Laser Photonics Reviews.

[98] Pitchappa P., Kumar A., Prakash S., Jani H., Venkatesan T., Singh R. (2019). Chalcogenide Phase Change Material for Active Terahertz Photonics. Adv. Mater..

[99] Kodama C.H., Coutu R.A. (2016). Tunable Split-Ring Resonators Using Germanium Telluride. Appl. Phys. Lett..

[100] Gwin A.H., Kodama C.H., Laurvick T.V., Coutu R.A., Taday P.F. (2015). Improved Terahertz Modulation Using Germanium Telluride (GeTe) Chalcogenide Thin Films. Appl. Phys. Lett..

[101] Lin Q.W., Wong H., Huitema L., Crunteanu A. (2021). Coding Metasurfaces with Reconfiguration Capabilities Based on Optical Activation of Phase-Change Materials for Terahertz Beam Manipulations. Adv. Opt. Mater..

[102] Savo S., Shrekenhamer D., Padilla W.J. (2014). Liquid Crystal Metamaterial Absorber Spatial Light Modulator for THz Applications. Adv. Opt. Mater..

[103] Vasic B., Isic G., Beccherelli R., Zografopoulos D.C. (2020). Tunable Beam Steering at Terahertz Frequencies Using Reconfigurable Metasurfaces Coupled with Liquid Crystals. IEEE J. Sel. Top. Quantum Electron..

[104] Buchnev O., Podoliak N., Kaltenecker K., Walther M., Fedotov V.A. (2020). Metasurface-Based Optical Liquid Crystal Cell as an Ultrathin Spatial Phase Modulator for THz Applications. ACS Photonics.

[105] Wu J., Shen Z., Ge S., Chen B., Shen Z., Wang T., Zhang C., Hu W., Fan K., Padilla W. (2020). Liquid Crystal Programmable Metasurface for Terahertz Beam Steering. Appl. Phys. Lett..

[106] Liu C.X., Yang F., Fu X.J., Wu J.W., Zhang L., Yang J., Cui T.J. (2021). Programmable Manipulations of Terahertz Beams by Transmissive Digital Coding Metasurfaces Based on Liquid Crystals. Adv. Opt. Mater..

[107] Oberhammer J. THz MEMS-Micromachining Enabling New Solutions at Millimeter and Submillimeter Frequencies. Proceedings of the 2016 Global Symposium on Millimeter Waves, GSMM 2016 and ESA Workshop on Millimetre-Wave Technology and Applications.

[108] Kappa J., Sokoluk D., Klingel S., Shemelya C., Oesterschulze E., Rahm M. (2019). Electrically Reconfigurable Micromirror Array for Direct Spatial Light Modulation of Terahertz Waves over a Bandwidth Wider Than 1 THz. Sci. Rep..

[109] Cong L., Pitchappa P., Wu Y., Ke L., Lee C., Singh N., Yang H., Singh R. (2017). Active Multifunctional Microelectromechanical System Metadevices: Applications in Polarization Control, Wavefront Deflection, and Holograms. Adv. Opt. Mater..

[110] Manjappa M., Pitchappa P., Singh N., Wang N., Zheludev N.I., Lee C., Singh R. (2018). Reconfigurable MEMS Fano Metasurfaces with Multiple-Input–Output States for Logic Operations at Terahertz Frequencies. Nat. Commun..

[111] Shen Y., Wang J., Wang Q., Qiao X., Wang Y., Xu D. (2021). Broadband Tunable Terahertz Beam Deflector Based on Liquid Crystals and Graphene. Crystals.

[112] Li H., Xu W., Cui Q., Wang Y., Yu J. (2020). Theoretical Design of a Reconfigurable Broadband Integrated Metamaterial Terahertz Device. Opt. Express.

[113] Chen K., Zhang X., Chen X., Wu T., Wang Q., Zhang Z., Xu Q., Han J., Zhang W. (2021). Active Dielectric Metasurfaces for Switchable Terahertz Beam Steering and Focusing. IEEE Photonics J..

[114] Zhang W., Zhang B., Fang X., Cheng K., Chen W., Wang Z., Hong D., Zhang M. (2021). Microfluid-Based Soft Metasurface for Tunable Optical Activity in THz Wave. Opt. Express.

[115] Caldwell J.D., Lindsay L., Giannini V., Vurgaftman I., Reinecke T.L., Maier S.A., Glembocki O.J. (2015). Low-Loss, Infrared and Terahertz Nanophotonics Using Surface Phonon Polaritons. Nanophotonics.

[116] Basov D.N., Fogler M.M., García De Abajo F.J. (2016). Polaritons in van Der Waals Materials. Science.

[117] Low T., Chaves A., Caldwell J.D., Kumar A., Fang N.X., Avouris P., Heinz T.F., Guinea F., Martin-Moreno L., Koppens F. (2017). Polaritons in Layered Two-Dimensional Materials. Nat. Mater..

[118] Dai Z., Hu G., Ou Q., Zhang L., Xia F., Garcia-Vidal F.J., Qiu C.W., Bao Q. (2020). Artificial Metaphotonics Born Naturally in Two Dimensions. Chem. Rev..

[119] Ma W., Shabbir B., Ou Q., Dong Y., Chen H., Li P., Zhang X., Lu Y., Bao Q. (2020). Anisotropic Polaritons in van Der Waals Materials. InfoMat.

[120] Song M., Jayathurathnage P., Zanganeh E., Krasikova M., Smirnov P., Belov P., Kapitanova P., Simovski C., Tretyakov S., Krasnok A. (2021). Wireless Power Transfer Based on Novel Physical Concepts. Nat. Electron..

[121] Ni G.X., McLeod A.S., Sun Z., Wang L., Xiong L., Post K.W., Sunku S.S., Jiang B.Y., Hone J., Dean C.R. (2018). Fundamental Limits to Graphene Plasmonics. Nature.

[122] Alonso-González P., Nikitin A.Y., Gao Y., Woessner A., Lundeberg M.B., Principi A., Forcellini N., Yan W., Vélez S., Huber A.J. (2017). Acoustic Terahertz Graphene Plasmons Revealed by Photocurrent Nanoscopy. Nat. Nanotechnol..

[123] Walsh B.M., Foster J.C., Erickson P.J., Sibeck D.G. (2014). Tunable Phonon Polaritons in Atomically Thin van Der Waals Crystals of Boron Nitride. Science.

[124] Ma W., Alonso-González P., Li S., Nikitin A.Y., Yuan J., Martín-Sánchez J., Taboada-Gutiérrez J., Amenabar I., Li P., Vélez S. (2018). In-Plane Anisotropic and Ultra-Low-Loss Polaritons in a Natural van Der Waals Crystal. Nature.

[125] Zheng Z., Xu N., Oscurato S.L., Tamagnone M., Sun F., Jiang Y., Ke Y., Chen J., Huang W., Wilson W.L. (2019). A Mid-Infrared Biaxial Hyperbolic van Der Waals Crystal. Sci. Adv..

[126] Wu Y., Ou Q., Yin Y., Li Y., Ma W., Yu W., Liu G., Cui X., Bao X., Duan J. (2020). Chemical Switching of Low-Loss Phonon Polaritons in α-MoO3 by Hydrogen Intercalation. Nat. Commun..

[127] Taboada-Gutiérrez J., Álvarez-Pérez G., Duan J., Ma W., Crowley K., Prieto I., Bylinkin A., Autore M., Volkova H., Kimura K. (2020). Broad Spectral Tuning of Ultra-Low-Loss Polaritons in a van Der Waals Crystal by Intercalation. Nat. Mater..

[128] Wu Y., Ou Q., Dong S., Hu G., Si G., Dai Z., Qiu C.W., Fuhrer M.S., Mokkapati S., Bao Q. (2021). Efficient and Tunable Reflection of Phonon Polaritons at Built-In Intercalation Interfaces. Adv. Mater..

[129] Hu G., Ou Q., Si G., Wu Y., Wu J., Dai Z., Krasnok A., Mazor Y., Zhang Q., Bao Q. (2020). Topological Polaritons and Photonic Magic Angles in Twisted α-MoO3 Bilayers. Nature.

[130] Dai S., Ma Q., Liu M.K., Andersen T., Fei Z., Goldflam M.D., Wagner M., Watanabe K., Taniguchi T., Thiemens M. (2015). Graphene on Hexagonal Boron Nitride as a Tunable Hyperbolic Metamaterial. Nat. Nanotechnol..

[131] Zhang Q., Ou Q., Hu G., Liu J., Dai Z., Fuhrer M.S., Bao Q., Qiu C.W. (2021). Hybridized Hyperbolic Surface Phonon Polaritons at α-MoO3and Polar Dielectric Interfaces. Nano Lett..

[132] Álvarez-Pérez G., González-Morán A., Capote-Robayna N., Voronin K.V., Duan J., Volkov V.S., Alonso-González P., Nikitin A.Y. (2022). Active Tuning of Highly Anisotropic Phonon Polaritons in Van Der Waals Crystal Slabs by Gated Graphene. ACS Photonics.

[133] Zeng Y., Ou Q., Liu L., Zheng C., Wang Z., Gong Y., Liang X., Zhang Y., Hu G., Yang Z. (2022). Tailoring Topological Transition of Anisotropic Polaritons by Interface Engineering in Biaxial Crystals. arXiv.

[134] Huang C.X., Zhang J., Cheng Q., Cui T.J. (2021). Polarization Modulation for Wireless Communications Based on Metasurfaces. Adv. Funct. Mater..

[135] Chen X., Ke J.C., Tang W., Chen M.Z., Dai J.Y., Basar E., Jin S., Cheng Q., Cui T.J. (2021). Design and Implementation of MIMO Transmission Based on Dual-Polarized Reconfigurable Intelligent Surface. IEEE Wirel. Commun. Lett..

[136] Wong H., Wang K.X., Huitema L., Crunteanu A. (2020). Active Meta Polarizer for Terahertz Frequencies. Sci. Rep..

[137] Nakanishi T., Nakata Y., Urade Y., Okimura K. (2020). Broadband Operation of Active Terahertz Quarter-Wave Plate Achieved with Vanadium-Dioxide-Based Metasurface Switchable by Current Injection. Appl. Phys. Lett..

[138] Zhang M., Zhang W., Liu A.Q., Li F.C., Lan C.F. (2017). Tunable Polarization Conversion and Rotation Based on a Reconfigurable Metasurface. Sci. Rep..

[139] Lee W.S.L., Nirantar S., Headland D., Bhaskaran M., Sriram S., Fumeaux C., Withayachumnankul W. (2018). Broadband Terahertz Circular-Polarization Beam Splitter. Adv. Opt. Mater..

[140] Castaldi G., Zhang L., Moccia M., Hathaway A.Y., Tang W.X., Cui T.J., Galdi V. (2021). Joint Multi-Frequency Beam Shaping and Steering via Space–Time-Coding Digital Metasurfaces. Adv. Funct. Mater..

